# Recalibrating expectations about effect size: A multi-method survey of effect sizes in the ABCD study

**DOI:** 10.1371/journal.pone.0257535

**Published:** 2021-09-23

**Authors:** Max M. Owens, Alexandra Potter, Courtland S. Hyatt, Matthew Albaugh, Wesley K. Thompson, Terry Jernigan, Dekang Yuan, Sage Hahn, Nicholas Allgaier, Hugh Garavan

**Affiliations:** 1 Department of Psychiatry, University of Vermont, Burlington, VT, United States of America; 2 Psychology Department, University of Georgia, Athens, GA, United States of America; 3 Division of Biostatistics, Department of Family Medicine and Public Health, University of California, San Diego, La Jolla, CA, United States of America; 4 University of California, San Diego, CA, United States of America; Indiana University, UNITED STATES

## Abstract

Effect sizes are commonly interpreted using heuristics established by Cohen (e.g., small: *r* = .1, medium *r* = .3, large *r* = .5), despite mounting evidence that these guidelines are mis-calibrated to the effects typically found in psychological research. This study’s aims were to 1) describe the distribution of effect sizes across multiple instruments, 2) consider factors qualifying the effect size distribution, and 3) identify examples as benchmarks for various effect sizes. For aim one, effect size distributions were illustrated from a large, diverse sample of 9/10-year-old children. This was done by conducting Pearson’s correlations among 161 variables representing constructs from all questionnaires and tasks from the Adolescent Brain and Cognitive Development Study® baseline data. To achieve aim two, factors qualifying this distribution were tested by comparing the distributions of effect size among various modifications of the aim one analyses. These modified analytic strategies included comparisons of effect size distributions for different types of variables, for analyses using statistical thresholds, and for analyses using several covariate strategies. In aim one analyses, the median in-sample effect size was .03, and values at the first and third quartiles were .01 and .07. In aim two analyses, effects were smaller for associations across instruments, content domains, and reporters, as well as when covarying for sociodemographic factors. Effect sizes were larger when thresholding for statistical significance. In analyses intended to mimic conditions used in “real-world” analysis of ABCD data, the median in-sample effect size was .05, and values at the first and third quartiles were .03 and .09. To achieve aim three, examples for varying effect sizes are reported from the ABCD dataset as benchmarks for future work in the dataset. In summary, this report finds that empirically determined effect sizes from a notably large dataset are smaller than would be expected based on existing heuristics.

## Introduction

In its contemporary usage, the term “effect size” refers to a standardized index of the strength or magnitude of an association between two variables or the size of difference between two groups [1, 2]. Although effect sizes are increasingly important in social science discourse, their interpretation has, in many cases, not advanced beyond the heuristics proposed by Jacob Cohen [3, 4], despite his admission that these heuristics were arbitrarily chosen. Cohen proposed that a Pearson’s correlation of *r* = .5 (or a Cohen’s *d* = .8) constitutes a “large” effect, *r* = .3 (*d* = .5) a medium effect, and *r* = .1 (*d* = .3) a small effect. More recently, there have been some efforts to reinterpret the meaning of these effect size estimates. In their excellent review of effect sizes, Funder and Ozer [5] suggest that even very small effects (i.e., *r* = .05) can accumulate over time and that effect sizes should be evaluated with regard to both short- and long-term cumulative impacts.

To improve our understanding of effect sizes, one strategy is to interpret magnitude of effects through a lens of common experiences. For example, Meyer and colleagues [6] lay out benchmarks for effects of various sizes helping researchers develop better concrete understanding about effect sizes using examples from daily life (e.g., antihistamine use and runny nose, *r* = .11). Another approach to improving our conceptualization of effect sizes is to consider the distribution of effect sizes observed across a broad range of social science applications. One recent meta-analysis from six prominent psychology journals found that the average published effect size was *r* = .19 and that the 90^th^ percentile of effect sizes was only *r* = .41 [7]. Another meta-analysis of nearly 1,000 published studies across sub-disciplines of psychological research found a median effect size of *r* = .16 for pre-registered studies, whereas studies that were not pre-registered had a median effect size of *r* = .36 [8]. Additionally, low power is an issue that permeates psychological research and its adjacent fields and is known to create over-estimates of effect size, operating through what is called the “winner’s curse” [9–11]. This suggests there are numerous processes related to the conduct and publication of psychological research that are likely creating artificially high effect sizes throughout the psychological literature.

In the current study, the first goal was to describe the distribution of effect sizes found in the questionnaire and task data from the Adolescent Brain Cognitive Development℠ Study (ABCD; www.ABCDstudy.org) using a common and well understood metric of effect size, Pearson’s correlation. The second goal was to qualify how various types of data and analytic approaches affect the distribution of effect size. The third goal was to provide concrete examples of different effect sizes (i.e., correlations) found in this dataset. This was done to illustrate the magnitudes and patterns of effect sizes that are likely to be observed in psychology/psychiatry research generally. The ABCD study is uniquely suited to address these goals because 1) it has a very large sample (*N* = 11,875) that approximates the demographic diversity of the current U.S. population [12–14], allowing for more accurate estimation of “true” effect sizes; 2) it contains extensive, multi-method data, allowing for specific quantification of how effect sizes differ across various scenarios (e.g., within and across reporters); 3) it contains pre-adolescent children, a group that is under-represented in meta-science research on effect size.

## Methods

### Procedures & participants

The current analysis was categorized as non-human research by the University of Vermont Institutional Review Board since it uses only de-identified archival data. However, the ABCD study itself was approved by the Institutional Review Board of University of California San Diego (IRB# 160091) and all ABCD data collection sites were approved by their respective Institutional Review Boards. Written consent was obtained from parents and written assent was obtained from participants.

The ABCD Study is an ongoing multi-site, longitudinal study following a cohort of 11,875 youths over ten years. Participants were recruited at ages 9 or 10 from 22 sites around the United States. They are followed for 10 years with questionnaire and neuropsychological task data collected in an office visit every year and neuroimaging data collected every two years. In this analysis, data were used from the baseline visits at which participants were 9 or 10 years old. All data used in the current study were collected at a single visit or across two visits that occurred within 30 days of each other. Participants with any valid data at the ABCD baseline visit were included in the analysis. Pairwise deletion was used (i.e., dropping subjects only from those correlations in which they were missing data for one or both variables). 90% of the correlations tested included data for over 10,000 participants and the smallest sample for any correlation test was 3,276.”

### Measures

The ABCD Study battery assessed a wide range of constructs including psychopathology, cognitive function, personality, childhood/prenatal development, social and extracurricular functioning, and demographic information. These previously validated instruments include the Child Behavior Checklist [15], the Urgency, Premeditation, Perseverance, Sensation Seeking, and Positive Urgency (UPPS-P) impulsive behavior scale [16], the Vancouver Index of Acculturation [17], among others. Some instruments contained multiple scales assessing unique constructs (e.g., the five UPPS-P scales). Some instruments assess multiple constructs with only a single item scale (e.g., demographic scale assesses child age, child sex, and child race with a single item for each). In several cases, composite scores were created for infrequently endorsed single item scales (e.g., individual prenatal/birthing complications were aggregated into a single variable).

Variables were selected for as many instruments/scales as possible to create the broadest estimate of average effect sizes. Specific goals in selecting variables were 1) to include at least one scale (e.g., the aggressive behavior scale of the Child Behavior Checklist) from each instrument (e.g., the Child Behavior Checklist) administered in the ABCD study, 2) to include as many non-overlapping scales as possible from each instrument, 3) to include a scale capturing each construct (e.g., aggression) assessed in the ABCD study, and 4) to exclude scales that contained overlapping items—for example, the externalizing composite scale from the Child Behavior Checklist was not used, since it is the summation of the aggressive behavior, rule-breaking, and intrusiveness scales. To assemble the list one author reviewed the instrument and variable lists available at https://nda.nih.gov/data_dictionary.html?source=ABCDALL and created a preliminary list of variables. This list was reviewed for completeness by another author familiar with the battery of the ABCD Study. This author made suggestions for any constructs assessed by the ABCD Study that were not captured by the list, which were subsequently added as new variables. For scales without pre-existing summary scores, new summary scores were created, as described in S1 Table in S1 File. Ultimately, analyses included 161 variables (i.e., scales) derived from 33 instruments. See S2 and S3 Tables in S1 File for a list of the instruments and scales included in these analyses.

### Data analysis

#### Data analysis software and code

Analyses were primarily conducted in Python version 3.7.3 using the packages pandas, numpy, matplotlib, sklearn, and pingouin. The mixed effect analyses were conducted in R version 3.6.1 using gamm4 and MuMin. Code for all analyses is available at https://github.com/owensmax/Effect_Size.

#### Effect size descriptions

First, descriptive statistics were examined for the effect size distributions for associations between all scales regardless of whether the associations met a threshold for statistical significance. Effect size was measured using Pearson’s correlations with absolute values of these correlations being reported. Then, to examine how the use of statistical thresholds altered the observed effect sizes, these analyses were repeated thresholding the distribution of effect sizes using several statistical significance strategies. Effect sizes distributions were reported only for associations that were indicated as significant under a given alpha threshold. The thresholds examined were the Bonferroni correction (corrected for 12,880 tests), the Benjamini-Hochberg false discovery rate correction (corrected for 12,880 tests)[18], and uncorrected *p* < .05. Furthermore, to examine how the use of covariates affects the distributions of effect sizes, analyses were repeated using partial correlations that included six common sociodemographic covariates: age, sex, race, parent income, parent education, parent marital status, and scanner site. These covariates were chosen because they are the standard covariates available in the ABCD Data Exploration and Analysis Portal (https://deap.nimhda.org/applications/User/login.php). Family was not accounted for in these analyses. In addition, analyses were repeated using mixed effects models to account for scanner site and sibling status (i.e., family id) as nested random effects (1|site/family id), with age, sex, race, parent income, parent education, and parent marital status as fixed effect covariates. From this, marginal pseudo R^2^ was derived and its square root taken to create an equivalent to Pearson’s correlation. These statistical threshold and covariate analyses were done to describe how various data analytic strategies that are common in psychological research modify the observed distribution of effect sizes. Specifically, they were designed to investigate the impact of adding common covariates to effect size estimates (e.g., how does covarying for age in associational analyses impact effect sizes?) and the consequence of statistical thresholding (e.g., how does thresholding associations using a Bonferroni correction alter observed effect sizes?).

To examine what factors might systematically influence effect sizes, associations were examined using several grouping schemes: 1) within instrument or between instrument (i.e., two scales from the same questionnaire vs. different questionnaires), 2) within domain or between domain (e.g., neurocognitive tests—questionnaires vs. questionnaires—questionnaires), 3) within reporter or between reporter (e.g., child self-report—child self-report vs. child self-report—parent-report). For the domain analyses, the domains used were mental health, sociodemographic, biological, cognitive task, personality, and social/family. To assess the relative importance of the different grouping schemes, a series of multiple regressions were conducted testing whether different grouping schemes would predict effect sizes in separate and combined regression models. In these analyses, each pairwise correlation was converted to a z-score. Additionally, each correlation was assigned a 1 or a 0 for three variables indicating whether the correlation was 1) within- or between-instrument, 2) within- or between-domain, and 3) within- or between-reporter. Each of these grouping variables was first tested separately as a predictor of effect size and then all were tested simultaneously in a multiple regression to assess the relative importance of each grouping. This procedure is outlined in detail in S4 Table in S1 File.

Next, distribution of effect sizes was examined under conditions typical of “real-world” analyses that are likely to be conducted with the ABCD dataset. In these analyses, associations were derived from mixed effects models with site and family as random effects, corrected for the six sociodemographic covariates as fixed effects, thresholded using the false discovery rate correction, and limited to only associations between scales coming from different instruments. Then, in a supplementary analysis, all analyses were repeated using Spearman’s rank order correlation to test if the choice of Pearson’s correlation was biasing results. Finally, to address the concern that effect sizes might be artificially deflated by low internal reliability for some abbreviated scales administered in the ABCD Study, Cronbach’s alpha was calculated for scales for which it was possible (*n* = 53; listed in S5 Table in S1 File). Then, for each association that included two of these scales, the product of the two alphas was calculated and the association between this product and the strength of the correlation between the two scales was assessed. This analysis provided insight into the extent to which observed effects size when associating two scales was related to each scale’s internal reliability.

## Results

**Fig 1:**
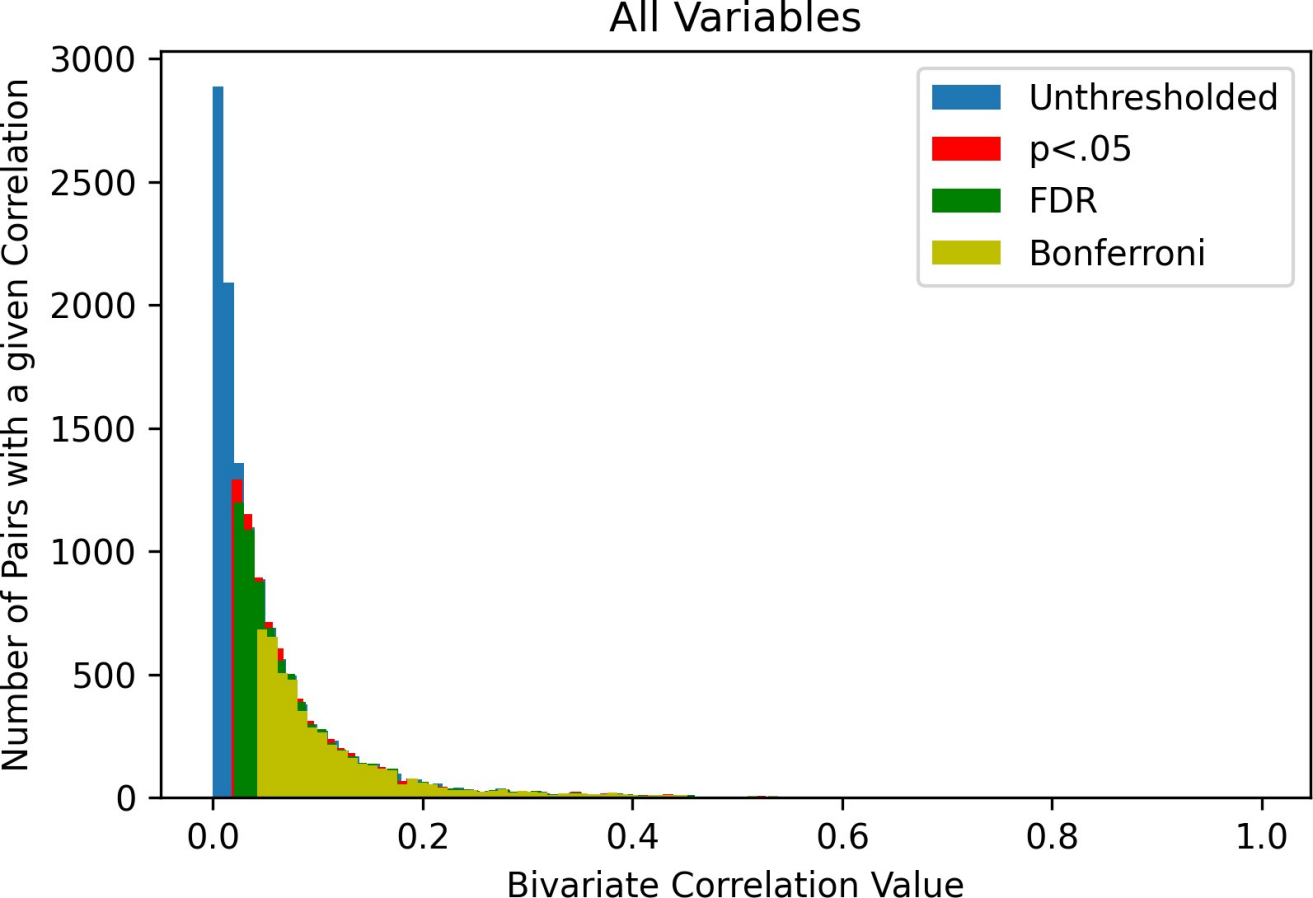
Effect size distributions at multiple statistical thresholds for non-covaried associations. FDR = false discovery rate.

The distribution of correlations across the entirety of the 161 instruments is reported in Table 1 and depicted in Fig 1. This distribution was heavily positively skewed with a first quartile of *r* = .01, median of *r* = .03, third quartile of *r* = .07, and 90^th^ percentile of *r* = .14. In examining differences in analyses when only one member of each family was used (i.e., removing the influence of siblings), results were essentially identical to the full sample analyses (Q1/Med/Q3/90^th^ *r* = .01/.03/.07/.14).

**Table 1:** Effect size (in Pearson’s r) quantiles for analytic variations.

Quantile	.1	.25	.5	.75	.9
**All Correlations**	.00	.01	.03	.07	.14
**Within Instrument**	.00	.01	.04	.14	.38
**Between Instrument**	.00	.01	.03	.07	.13
**Within Reporter**	.00	.01	.04	.10	.21
**Between Reporter**	.00	.01	.03	.06	.11
**Within Domain**	.01	.02	.06	.16	.30
**Between Domain**	.00	.01	.03	.06	.11
***p* < .05**	.02	.03	.06	.10	.18
**FDR**	.03	.04	.06	.11	.18
**Bonferroni**	.05	.06	.09	.14	.23
**Partial Correlation**	.00	.01	.02	.04	.10
**Mixed Effects Modeling**	.00	.01	.02	.04	.10
**“Real-World”**	.03	.03	.05	.09	.18

Within Reporter = correlations among variables derived from the reporting of the same individual (e.g., both variables based on parent-report measures); Between Reporter = correlations among variables derived from the reporting of different individuals (e.g., one variable based on parent report and the other based on child report).

**Fig 2:**
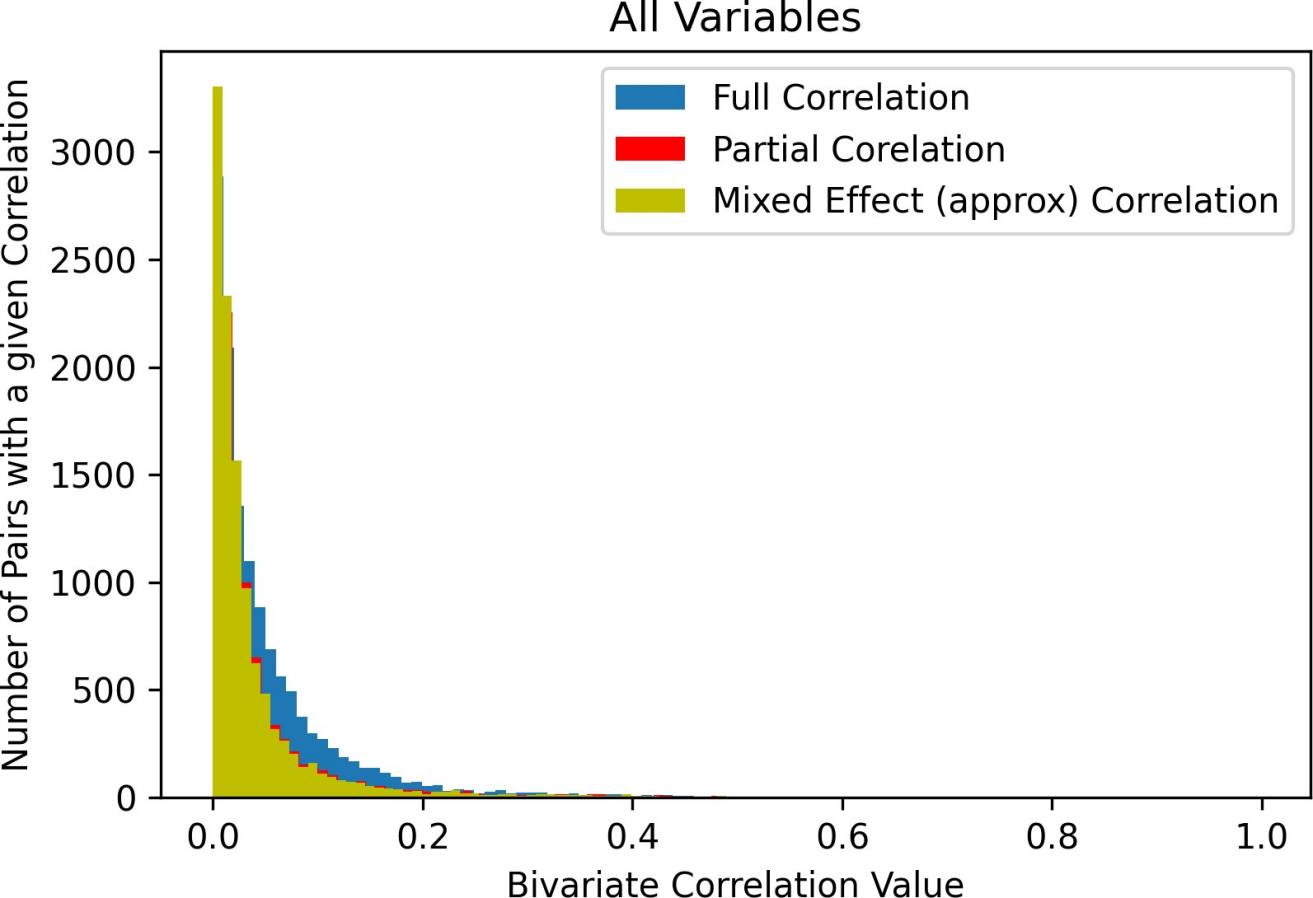
In yellow: Distribution of correlations from mixed effect modeling controlling for age, sex, race, parent income, parent education, parent marital status, site (as a random effect), and family id (as a random effect). In red: partial correlations controlling for age, sex, race, parent income, parent education, parent marital status, and site (in red). In blue: bivariate Pearson correlations.

When statistical significance thresholding was applied to these analyses, a similar pattern emerged for all levels of thresholding, although shifted increasingly positively as the stringency of thresholding increased (Fig 1). Even under the most stringent threshold (i.e., Bonferroni), the median effect size remained low (*r* = .09) and the 90^th^ percentile was *r* = .23. When analyses were repeated using partial correlations to control for age, sex, race, parent income, parent education, parent marital status, and site, the distribution of effect sizes was similar to bivariate associations (see Fig 2), albeit somewhat smaller in magnitude. When analyses were repeated using mixed effects models to control for site and family as random effects, results were essentially identical to partial correlations.

**Fig 3:**
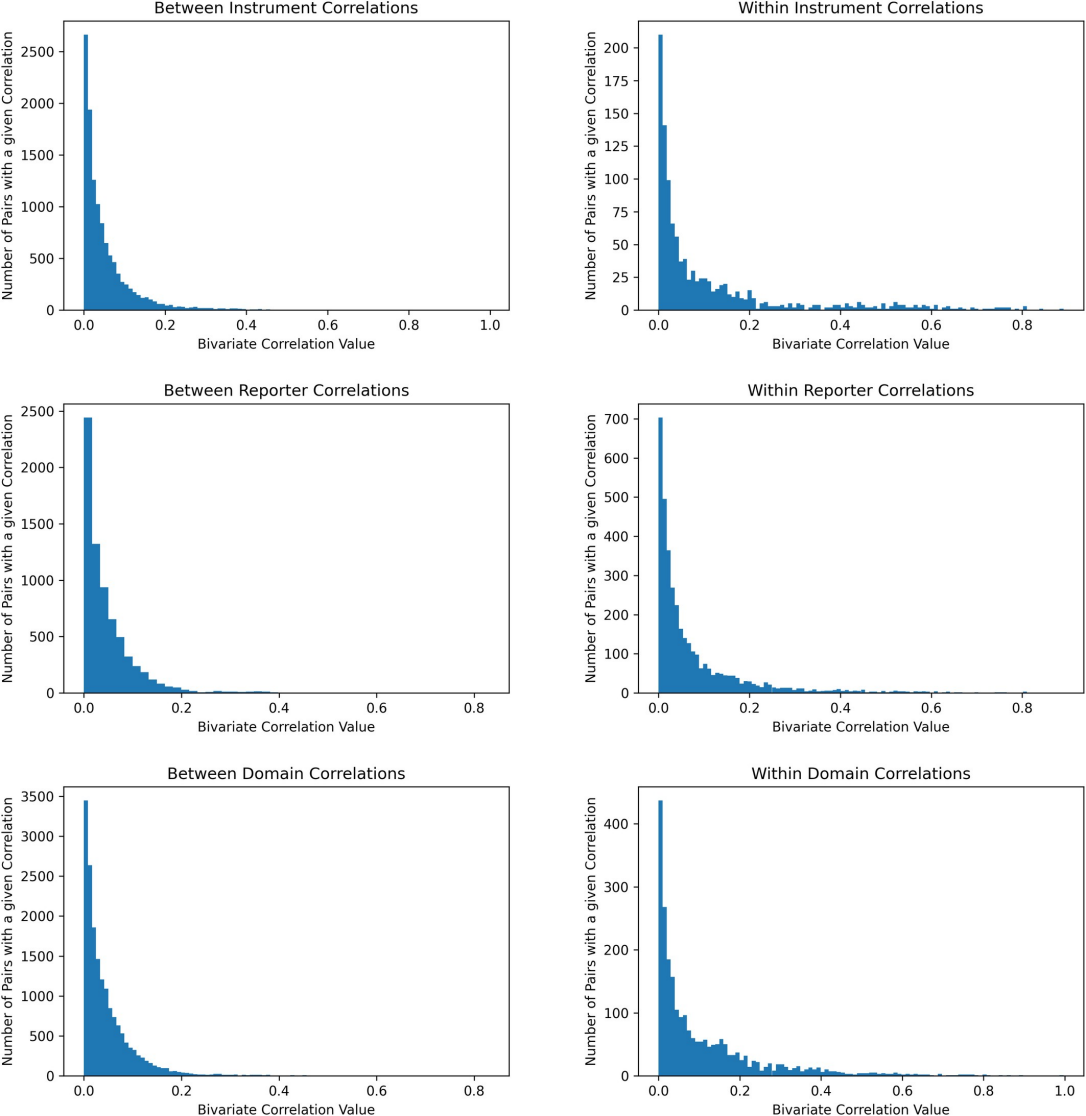
Qualifications of effect size distribution.

**Fig 4:**
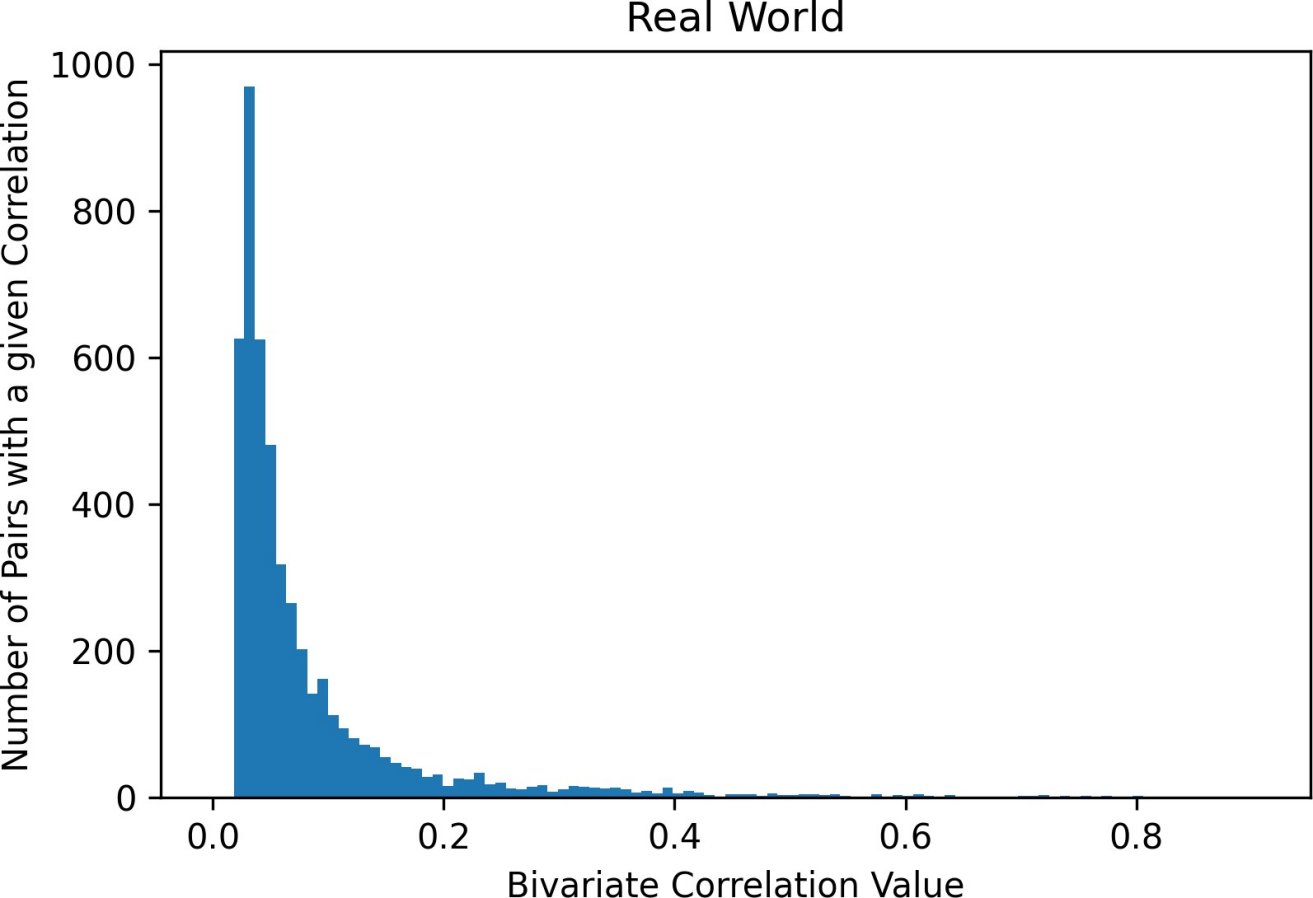
Distribution of effect sizes under “real-world” conditions (mixed effect model corrected for site, family, and sociodemographic covariates, thresholded using a false discovery rate correction, and limited to only associations between scales coming from different instruments).

When associations were split into those occurring between two different scales from the same instrument and two scales from two different instruments, scales from the same instrument showed relatively larger correlations (*p* < 2e-16; Median *r* = .04 vs .03, 90^th^ percentile *r* = .38 vs .13; Fig 4). Likewise, when associations were split into those of scales from within the same domain or those from separate domains, same-domain associations showed larger effect sizes than different-domain associations (*p* < 2e-16; Median *r* = .06 vs .03, 90^th^ percentile *r* = .30 vs .11). Furthermore, when associations were split into those in which the reporters of the two scales were the same or those in which the reporters of the two scales were different, same-reporter associations showed larger effect sizes than different reporter associations (*p* < 2e-16; Median *r* = .04 vs .03, 90^th^ percentile *r* = .21 vs .11). Histograms of the distributions of these comparisons are found in Fig 3. When all three grouping schemes were compared in simultaneous regression, both within-domain (*p* < 2e-16) and within-reporter (*p* = 5e-8) associations were significantly larger than between-domain and between-reporter associations. However, within instrument associations did not differ from between instrument associations after accounting for domain and reporter (*p* = .98).

In “real-world” analyses (mixed effect model corrected for the sociodemographic covariates, thresholded using the false discovery rate correction, and limited to only associations between scales coming from different instruments; see Fig 4), the median effect size was .05, first and third quartile were .03 and .09, and the 90^th^ percentile was .18.

When primary analyses were repeated using Spearman’s correlation, results were mostly identical, except for analyses using partial correlations, which were meaningfully elevated (i.e., approximately twice as large; S6 Table in S1 File). When examining the association of reliability with effect size, a small, positive association was found between scale internal reliability (i.e., Cronbach’s alpha) with correlation magnitude (*r* = .09, *p* = .02). The distribution of the Cronbach’s alphas was broadly Gaussian, with mean .72, Q1 = .66, Q3 = .81, 10th percentile = .59, 90th percentile = .88. This distribution is displayed in S1 Fig in S1 File.

## Discussion

The current report describes the distribution of effect sizes throughout the questionnaire and task data collected as part of the ABCD study. Based on this analysis, most effect sizes within the ABCD data would be labeled by Cohen’s heuristic as “small” and effect sizes that would be labeled “medium” or “large” were uncommon. Many associations which a typical, informed reader would intuitively have expected to be “medium” or “large” were below *r* = .20, such as attention problems and impulsivity, age and pubertal development, and child and familial history of psychiatric problems.

Several factors that might influence effect sizes were examined, including the consequences of correlating within and across instruments, within and across content domains, and within and across reporters. As expected, in all cases within domain/instrument/reporter associations were larger than between domain/instrument/reporter associations. Given that the majority of research questions in contemporary psychology/psychiatry tend to explore the relationship between distinct constructs (e.g., two variables occurring at different levels of analysis), it is likely that the between domain/instrument/reporter effect size distributions are more representative of effect sizes that would be observed in most contemporary research. At the very least, essentially all questions being explored involve associations between two different instruments, making the “between instrument” distribution a bare minimum for consideration as a realistic distribution of effect sizes. Additionally, analyses were conducted using several significance thresholds changed the distribution of effect sizes. While the median effect size did increase when statistical thresholds were used, even using the most stringent threshold (Bonferroni correction), the median effect size was still less than *r* = .10 (Q1/Q2/Q3 = .06/.09/.14). While it is not applicable to all research questions, most studies do make some attempt to rule out confounding variables such as participant’s age, sex, and socioeconomic status. This approach was mirrored in the current study by covarying for six common sociodemographic covariates. Results of this version of this analysis were similar to the basic analyses, though adding covariates did slightly lower the median and ceiling of effect sizes seen. In sum, the current results suggest a potential new heuristic for bivariate effect sizes based on the 25^th^, 50^th^, 75^th^, and 90^th^ percentiles of correlations in the ABCD study: a “below average” effect size is around .03, an “average” effect size is one of around .05, an “above average” effect size is one of around .09, and an “extremely above average” effect size is one around .18 and above. However, this suggested heuristic is less likely to be appropriate as the design of a study diverges from that of the ABCD study. In other words, this heuristic is likely well suited for multi-site, multi-method correlational studies of children in the western world and less well suited for studies that do not share aspects of this methodological framework. At the least, these heuristics should be considered well-founded for future work conducted in the ABCD study and studies like it.

The current report provides examples of various effect sizes as benchmarks in Table 2. While most correlations described as “very large” are between two variables that are either A) measured by the same scale (e.g., Positive Urgency and Negative Urgency, fluid intelligence and crystallized intelligence) or B) highly interrelated physical properties (e.g., height and weight), there are some very large effects that do represent clinically “interesting” associations, such as stress and sleep problems or attention problems and impulsivity. However, it should be noted that there are numerous correlations that are smaller despite being highly intuitive (e.g., sleep problems and total cognitive ability; physical activity and weight), suggesting that intuition tends to over-estimate effect sizes.

**Table 2:** Effect size benchmarks.

	Variable 1	Variable 2	*r*
**Extremely Above Average**	Height	Weight	.60
	Parent total psychiatric problem	Total psychiatric problems	.57
	Stress	Sleep problems	.55
	Fluid intelligence	Crystallized intelligence	.48
	Age	Height	.43
	School performance	Reading ability	.40
	Aggressive behavior	Prosocial behavior	-.34
	Age	Weight	.24
	Attention problems	UPPS lack of perseverance	.22
	Traumatic experiences	Total psychological problems	.20
**Above Average**	Age	Pubertal development	.17
	Weight	Screen time	.16
	Family history of psychiatric problems	Total psychiatric problems	.15
	Total psychiatric problems	Total cognitive ability	-.14
	Flanker task performance	Attention problems	-.11
	Physical activity	Screen time	-.10
	Parental acceptance	Total psychiatric problems	-.09
**Average**	UPPS lack of premeditation	Detention frequency	.08
	Aggressive behavior	Flanker task performance	- .07
	Sleep problems	Total cognitive ability	-.06
	Family history of psychiatric problems	Prodromal psychosis symptoms	.05
**Below Average**	Caffeine consumption	Sleep problems	.04
	Physical activity	Weight	.03
	UPPS lack of premeditation	Total cognitive ability	-.02
	Age	Pro-social behavior	.01

All correlations are significant at *p* < .05. UPPS-P = Urgency, Premeditation, Perseverance, Sensation Seeking, and Positive Urgency Impulsive Behavior Scale. Extremely above average = 90^th^ percentile and above; above average = 75^th^– 89^th^ percentile; average = 50^th^ to 74^th^ percentile; below average = 49^th^ percentile and below.

One consideration is the generalizability of the current analyses to a typical study. The question could be raised “why examine all ABCD summary scores rather than testing only theoretically informed associations?”. Our reasoning for examining all associations was based on several factors. One, the ABCD instruments are all selected to address the central aim of understanding adolescent development and well-being and are therefore reasonably likely to be associated with each other. The widely accepted biopsychosocial model of disease and human development [19, 20] states that biological, psychological, and social forces (including cultural and spiritual aspects) interact in development to produce states of well-being and dysfunction (physical and mental). All the instruments used fall within the realm of biological, psychological, and social, indicating that the association of any two of these variables is a reasonable *a priori* hypothesis if the biopsychosocial model is invoked as a theoretical framework. Two, some scales were summarized to make variables more general (i.e., scales rather than single items where possible). For example, despite having no pre-existing summary score, summary scores were created from all the possible birth complications indicated on the developmental history questionnaire. This created a more general variable that represented the construct of birth complications generally, which was more likely to be related to other variables in the dataset. Thus, by aggregating items, sufficiently general variables were created that most have plausible associations amongst each other. Three, analyses were repeated using multiple significance thresholds so that only association pairs that were demonstrated to be statistically related to each other are used, finding largely similar results at all thresholds. Given the large sample size and strict threshold, there is no reason to think that associations found that exceed multiple comparison correction do not represent “true” associations. Four, even associations in 90^th^ percentile and above do not show effect sizes that would traditionally be considered large. For example, amongst false discovery rate corrected, between-instrument, mixed effect models (“real-world” analysis), the 90^th^ percentile of effect size was a correlation of .18 and the 95^th^ percentile was .29. Even if the lower half of the distribution was excessively noisy, the current analyses provide a valid ceiling of what constitutes an effect that researchers are likely to see “in the wild”. Five, the current results closely mirror the findings of Schaffer and Schwarz (2019), who found a positively skewed distribution of effect sizes (with similar median and interquartile range) among published studies that had been pre-registered.

Analyses on scale reliability showed a significant association between effect sizes and internal scale reliability, suggesting the size of effects in the current analysis was to some degree influenced by the reliability of the scales. This is relevant for the current analysis given that many of the instruments used in ABCD were short forms of more commonly used long-form instruments (e.g., the abbreviated UPPS-P for children). Thus, the use of more reliable, long-form instruments may likely increase effect size somewhat, but it should be noted that this effect seems unlikely to be substantial given the modest association of internal reliability with effect size seen in the current results.

There are several factors to consider when thinking about the effect sizes observed in this study. The effect sizes report associations of between-subject observational data and contain no within-subject or experimental components. Thus, the current estimates of typical effect sizes may not generalize to experimental or within-subject studies. Furthermore, the current analysis used only a univariate, linear modeling approach. While univariate analyses remain common, there is an increasing acceptance that multivariate approaches can be effective in explaining more variance in a psychological phenomenon. These results do not rule out the possibility that multivariate approaches may cumulatively achieve larger effect sizes. Likewise, this study does not rule out the possibility of larger non-linear effects or effects resulting from the interaction among multiple variables.

Thus, while there are factors worth considering about the specifics of the current analyses, the current results provide a useful demonstration in enhancing understanding of effect sizes in one of the largest and most comprehensive datasets in the fields of psychology/psychiatry. The current results suggest that effect sizes for self-report and cognitive task data collected from children aged 9–10 may be more consistent with recently proposed alternative heuristic schemes [5, 7, 8] than those Cohen previously suggested [3]. However, as Funder and Ozer [5] describe, effects perceived as small tend to accumulate across individuals and over time. Therefore, it is the opinion of the authors that the current results should not be interpreted as pessimistic about the future of psychological research, but rather they provide a necessary resetting of the expectation of finding elusive “large” effect sizes that have been conspicuously missing across recent big data initiatives. In the future, it is likely that the field would benefit from more open and candid discussions around the meaning ascribed to very small-to-small effects, given that these appear to be the rule for associations between psychological variables and not the exception.

## Supporting information

S1 File This contains all the supporting tables and figures (S1–S6 Tables and S1 Fig). (DOCX)
