# Supplementary material for: Recalibrating expectations about effect size: A multi-method survey of effect sizes in the ABCD study
Source: PLoS One. 2021 Sep 23;16(9):e0257535. doi: 10.1371/journal.pone.0257535 (PMC8460025; doi:10.1371/journal.pone.0257535)

**Supplemental Table 1.** Variables created to summarize measures without pre-existing summary scores. All variables not specified here come directly from Adolescent Brain and Cognitive Development Study RDS file.

| Measure | Name of variable created | Description of how variable created | Construct described by variable |
| --- | --- | --- | --- |
| Parent Acculturation Survey | accult_phenx_q45_p | Averages questions 4 and 5 of parent acculturation survey | Does parent speak English with friends/family? |
| Developmental History Questionnaire | devhx_distress_at_birth | Aggregates items 14a through 14h | Number of birth complications |
| Developmental History Questionnaire | devhx_milestones | Aggregates questions 19a, 19b, 19c, 19d | Number of developmental milestones met on time |
| Developmental History Questionnaire | devhx_mother_probs | Aggregates questions 10a through 10m | Number of mother health problems during pregnancy |
| Developmental History Questionnaire | devhx_ss_alcohol_avg_p | Aggregates questions 8 and 9 focused on use before and after knowing about pregnancy | Mother alcohol use during pregnancy |
| Developmental History Questionnaire | devhx_ss_alcohol_effects_p | Aggregates questions 8 and 9 focused on use before and after knowing about pregnancy | During pregnancy, how many drinks needed to feel effect? |
| Developmental History Questionnaire | devhx_ss_alcohol_max_p | Aggregates questions 8 and 9 focused on use before and after knowing about pregnancy | Most drinks drunk in one occasion while pregnant |
| Developmental History Questionnaire | devhx_ss_cigs_per_day_p | Aggregates questions 8 and 9 focused on use before and after knowing about pregnancy | Cigarettes per day during pregnancy |
| Developmental History Questionnaire | devhx_ss_coc_crack_amt_p | Aggregates questions 8 and 9 focused on use before and after knowing about pregnancy | Frequency Cocaine/Crack use during pregnancy |
| Developmental History Questionnaire | devhx_ss_marijuana_amt_p | Aggregates questions 8 and 9 focused on use before and after knowing about pregnancy | Frequency of Cannabis use during pregnancy |
| Developmental History Questionnaire | devhx_ss_oxycont_amt_p | Aggregates questions 8 and 9 focused on use before and after knowing about pregnancy | Frequency of Opiate Use During Pregnancy |
| Family History Assessment | famhx_total | Aggregates questions 7 through 13 | Family History of Mental Illness |
| Diagnostic Interview for DSM-5 Full (KSADS-5) | ksads_bipolar_composite | Aggregates items 830 through 839 | Child Report: Bipolar Diagnosis |
| Diagnostic Interview for DSM-5 Full (KSADS-5) | ksads_cd_composite | Aggregates items 897 through 900 | Child Report: Conduct Disorder Diagnosis |
| Diagnostic Interview for DSM-5 Full (KSADS-5) | ksads_depressive_comp | Aggregates items 840 through 846 | Child Report: Depressive Disorder Diagnosis |
| Diagnostic Interview for DSM-5 Full (KSADS-5) | ksads_eating_disorder_composite | Aggregates items 929 thorugh 944 | Child Report: Eating Disorder Diagnosis |
| Diagnostic Interview for DSM-5 Full (KSADS-5) | ksads_GAD_composite | Aggregates items 869, 870, 913, and 914 | Child Report: Generalized Anxiety Diagnosis |
| Diagnostic Interview for DSM-5 Full (KSADS-5) | ksads_nssi_composite | Aggregates items 945 and 956 | Child Report: Non-Suicidal Self Injury |
| Diagnostic Interview for DSM-5 Full (KSADS-5) | ksads_OCD_composite | Aggregates items 917, 918, and 919 | Child Report: Obsessive Compulsive Diagnosis |
| Diagnostic Interview for DSM-5 Full (KSADS-5) | ksads_psychosis_composite | Aggregates items 826, 827, 828, 829, 849, 850, 851, and 852 | Child Report: Psychotic Disorder Diagnosis |
| Diagnostic Interview for DSM-5 Full (KSADS-5) | ksads_ptsd_composite | Aggregates items 754 through 770 on ptsd section | Child Report: Post Traumatic Stress Diagnosis |
| Diagnostic Interview for DSM-5 Full (KSADS-5) | ksads_SAD_composite | Aggregates items 863, 864, 911, and 912 | Child Report: Social Anxiety Diagnosis |
| Diagnostic Interview for DSM-5 Full (KSADS-5) | ksads_sud_composite | Aggregates items 888, 889, 890, 893, and 894 | Child Report: Substance Use Disorder Diagnosis |
| Diagnostic Interview for DSM-5 Full (KSADS-5) | ksads_suicide_composite | Aggregates items 946 through 965 | Child Report: Suicide ideation, intent, or attempt |
| Medical History Questionnaire | medhx_er_composite | Aggregates questions 4b and 5b | Number of emergency room visits for child |
| Demographic | puberty | Average of parent & child: Pubertal Development | Pubertal development |
| Sports and Activities Involvement Questionnaire (SAIQ) | sports_activity_activities_p_ind_sport | Binary variable created if child participates in 1 or more “individual sport” activity on SAIQ | Participation in Individual Sports (e.g., tennis) |
| Sports and Activities Involvement Questionnaire (SAIQ) | sports_activity_activities_p_performance | Binary variable created if child participates in 1 or more “performance” activity on SAIQ | Participation in Performance Sports (e.g., dance) |
| Sports and Activities Involvement Questionnaire (SAIQ) | sports_activity_activities_p_team_sport | Binary variable created if child participates in 1 or more “team sport” activity on SAIQ | Participation in Team Sports (e.g., baseball) |
| Sports and Activities Involvement Questionnaire (SAIQ) | sports_activity_activities_p_hobbies | Binary variable created if child participates in 1 or more “hobby” activity on SAIQ | Participation in Hobbies (e.g., stamp collecting) |

**Supplemental Table 2.** Variables used in descriptive analysis of effect sizes.

| ABCD  MEASURE ID | DESCRIPTION  OF VARIABLE | INSTRUMENT | TYPE |
| --- | --- | --- | --- |
| accult_phenx_q1_p | How well parent speaks English | Parent Acculturation Survey | Single Item |
| accult_phenx_q2_p | Does parent speak second language? | Parent Acculturation Survey | Single Item |
| accult_phenx_q45_p | Does parent speak English with friends/family? | Parent Acculturation Survey | Average of 2 items |
| age | Age | Demographic | Single Item |
| anthro_height_calc | Measured Height | Youth Anthropometrics | Single Item |
| anthro_waist_cm | Measured Waist Size | Youth Anthropometrics | Single Item |
| anthro_weight_calc | Measured Weight | Youth Anthropometrics | Single Item |
| asr_scr_aggressive_r | Parent Aggression Symptoms | Achenbach Self Report, Parent | Multi-item scale |
| asr_scr_anxdep_r | Parent Anxious/Depressed Symptoms | Achenbach Self Report, Parent | Multi-item scale |
| asr_scr_attention_r | Parent Attention Problems | Achenbach Self Report, Parent | Multi-item scale |
| asr_scr_intrusive_r | Parent Intrusiveness | Achenbach Self Report, Parent | Multi-item scale |
| asr_scr_perstr_r | Parent Personal Strengths | Achenbach Self Report, Parent | Multi-item scale |
| asr_scr_rulebreak_r | Parent Rule-breaking | Achenbach Self Report, Parent | Multi-item scale |
| asr_scr_somatic_r | Parent Somatic Symptoms | Achenbach Self Report, Parent | Multi-item scale |
| asr_scr_thought_r | Parent Thought Problems | Achenbach Self Report, Parent | Multi-item scale |
| asr_scr_withdrawn_r | Parent Withdrawn/Depressed Symptoms | Achenbach Self Report, Parent | Multi-item scale |
| bisbas_ss_bas_drive | Behavioral Drive | Behavioral Inhibition/Behavioral Activation Scale | Multi-item scale |
| bisbas_ss_bas_fs | Fun Seeking | Behavioral Inhibition/Behavioral Activation Scale | Multi-item scale |
| bisbas_ss_bas_rr | Reward Responsiveness | Behavioral Inhibition/Behavioral Activation Scale | Multi-item scale |
| brain_injury_ss_agefirst_p | Age of first brain injury | Ohio State Traumatic Brain Injury Screen-Short Modified (OTBI) | Single Item |
| cbcl_scr_07_ocd_r | Obsessive compulsive symptoms | Child Behavior Checklist | Multi-item scale |
| cbcl_scr_07_sct_r | Sluggish Cognitive Tempo | Child Behavior Checklist | Multi-item scale |
| cbcl_scr_07_stress_r | Stress | Child Behavior Checklist | Multi-item scale |
| cbcl_scr_syn_aggressive_r | Aggressive Symptoms | Child Behavior Checklist | Multi-item scale |
| cbcl_scr_syn_anxdep_r | Anxious/Depressed Symptoms | Child Behavior Checklist | Multi-item scale |
| cbcl_scr_syn_attention_r | Attention Problems | Child Behavior Checklist | Multi-item scale |
| cbcl_scr_syn_rulebreak_r | Rule-breaking | Child Behavior Checklist | Multi-item scale |
| cbcl_scr_syn_social_r | Social Problems | Child Behavior Checklist | Multi-item scale |
| cbcl_scr_syn_somatic_r | Somatic Symptoms | Child Behavior Checklist | Multi-item scale |
| cbcl_scr_syn_thought_r | Thought Problems | Child Behavior Checklist | Multi-item scale |
| cbcl_scr_syn_withdep_r | Withdrawn/Depressed Symptoms | Child Behavior Checklist | Multi-item scale |
| crpbi_acceptance_ss_studycaregiver | Caregiver Acceptance | Children's Report of Parental Behavioral Inventory | Multi-item scale |
| demo_gender_id_p | Gender Identification | Demographic | Single Item |
| demo_prnt_age_p | Parent Age | Demographic | Single Item |
| demo_prnt_empl_p | Parent Employment | Demographic | Single Item |
| demo_prnt_empl_time_p | Parent Full/Part Time Employment | Demographic | Single Item |
| demo_prnt_ethn_p | Parent Ethnicity | Demographic | Single Item |
| demo_prnt_gender_id_p | Parent Gender ID | Demographic | Single Item |
| demo_prtnr_empl_p | Parent Partner Employment | Demographic | Single Item |
| demo_prtnr_empl_time_p | Parent Partner Employment Time | Demographic | Single Item |
| demo_relig_p | Religion | Demographic | Single Item |
| devhx_10_p | Did biological mother take vitamins during pregnancy | Developmental History Questionnaire | Single Item |
| devhx_11_times_med_prof_p | How many times did mother see doctor during pregnancy | Developmental History Questionnaire | Single Item |
| devhx_12a_born_premature_p | Premature Birth | Developmental History Questionnaire | Single Item |
| devhx_13_ceasarian_p | Cesarean Section | Developmental History Questionnaire | Single Item |
| devhx_15_days_incubator_p | Days in incubator after birth | Developmental History Questionnaire | Single Item |
| devhx_16_days_high_fever_p | Days has had a high fever during first year | Developmental History Questionnaire | Single Item |
| devhx_17_infections_serious_ill_p | Serious illnesses or infections in first year of life | Developmental History Questionnaire | Single Item |
| devhx_18_mnths_breast_fed_p | Month Breastfed | Developmental History Questionnaire | Single Item |
| devhx_2_birth_wt_lbs_p | Birthweight | Developmental History Questionnaire | Single Item |
| devhx_20_motor_dev_p | Speed of Motor Development | Developmental History Questionnaire | Single Item |
| devhx_21_speech_dev_p | Speed of Speech Development | Developmental History Questionnaire | Single Item |
| devhx_23b_age_wet_bed_p | Age Last Wet the Bed | Developmental History Questionnaire | Single Item |
| devhx_3_age_at_birth_mother_p | Age of Mother at Birth | Developmental History Questionnaire | Single Item |
| devhx_4_age_at_birth_father_p | Age of Father at Birth | Developmental History Questionnaire | Single Item |
| devhx_5_twin_p | Is a twin? | Developmental History Questionnaire | Single Item |
| devhx_6_pregnancy_planned_p | Was pregnancy that resulted in child planned? | Developmental History Questionnaire | Single Item |
| devhx_7_far_along_p | How far along was mother when she found out pregnant? | Developmental History Questionnaire | Single Item |
| devhx_caffeine_11_p | How much caffeine for mother during pregnancy? | Developmental History Questionnaire | Single Item |
| devhx_distress_at_birth | Number of birth complications | Developmental History Questionnaire | Multi-item scale |
| devhx_milestones | Number of milestones met on time | Developmental History Questionnaire | Multi-item scale |
| devhx_mother_probs | Number of mother health problems during pregnancy | Developmental History Questionnaire | Multi-item scale |
| devhx_ss_alcohol_avg_p | Mother alcohol use during pregnancy | Developmental History Questionnaire | Average of 2 items |
| devhx_ss_alcohol_effects_p | During pregnancy, how many drinks needed to feel effect? | Developmental History Questionnaire | Average of 2 items |
| devhx_ss_alcohol_max_p | Most drinks drunk in one occasion while pregnant | Developmental History Questionnaire | Average of 2 items |
| devhx_ss_cigs_per_day_p | Cigarettes per day during pregnancy | Developmental History Questionnaire | Average of 2 items |
| devhx_ss_coc_crack_amt_p | Frequency Cocaine/Crack use during pregnancy | Developmental History Questionnaire | Average of 2 items |
| devhx_ss_marijuana_amt_p | Frequency of Cannabis use during pregnancy | Developmental History Questionnaire | Average of 2 items |
| devhx_ss_oxycont_amt_p | Frequency of Opiate Use During Pregnancy | Developmental History Questionnaire | Average of 2 items |
| ehi_ss_score | Handedness | Youth Edinburgh Handedness Inventory Short Form (EHIS) | Multi-item scale |
| famhx_total | Family History of Mental Illness | Family History Assessment | Multi-item scale |
| fes_ss_fc | Family Conflict Child Report | Family Environment Scale-Family Conflict Subscale Modified from PhenX (FES) | Multi-item scale |
| fes_ss_fc_p | Family Conflict Parent Report | Family Environment Scale-Family Conflict Subscale Modified from PhenX (FES) | Multi-item scale |
| high.educ.bl | Parent Highest Education Level | Demographic | Single Item |
| hisp | Ethnicity | Demographic | Single Item |
| household.income.bl | Family Household Income | Demographic | Single Item |
| ksads_adhd_composite | Attention Disorder Diagnosis | Diagnostic Interview for DSM-5 Full (KSADS-5) | Single Item |
| ksads_back_c_best_friend_p | Parent Report: Does child have best friend? | Diagnostic Interview for DSM-5 Full (KSADS-5) | Single Item |
| ksads_back_c_bully_p | Parent Report: Is child bullied? | Diagnostic Interview for DSM-5 Full (KSADS-5) | Single Item |
| ksads_back_c_det_susp_p | Parent Report: Has child been suspended in past year? | Diagnostic Interview for DSM-5 Full (KSADS-5) | Single Item |
| ksads_back_c_drop_in_grades_p | Parent Report: Has child had drop in grades in past year? | Diagnostic Interview for DSM-5 Full (KSADS-5) | Single Item |
| ksads_back_c_gay_p | Parent Report: Child sexual orientation | Diagnostic Interview for DSM-5 Full (KSADS-5) | Single Item |
| ksads_back_c_gay_prob_p | Parent Report: Does child have problems from being gay? | Diagnostic Interview for DSM-5 Full (KSADS-5) | Single Item |
| ksads_back_c_how_well_school_p | Parent Report: How well is child doing in school? | Diagnostic Interview for DSM-5 Full (KSADS-5) | Single Item |
| ksads_back_c_mh_sa_p | Parent Report: Has child received mental health services? | Diagnostic Interview for DSM-5 Full (KSADS-5) | Single Item |
| ksads_back_c_school_setting_p | Parent Report: What type of school setting is child in (e.g., private, public) | Diagnostic Interview for DSM-5 Full (KSADS-5) | Single Item |
| ksads_back_c_trans_p | Is child transgender? | Diagnostic Interview for DSM-5 Full (KSADS-5) | Single Item |
| ksads_back_c_trans_prob_p | Parent Report: Parent Report: Does child have problems from being transgender? | Diagnostic Interview for DSM-5 Full (KSADS-5) | Single Item |
| ksads_back_conflict_p | Parent Report: Do parent and child have conflict? | Diagnostic Interview for DSM-5 Full (KSADS-5) | Single Item |
| ksads_back_grades_in_school_p | Parent Report: How are child’s grades in school? | Diagnostic Interview for DSM-5 Full (KSADS-5) | Single Item |
| ksads_back_det_susp | Child Report: Has child been suspended in past year? | Diagnostic Interview for DSM-5 Full (KSADS-5) | Single Item |
| ksads_back_drop_in_grades | Child Report: Has child had drop in grades in past year? | Diagnostic Interview for DSM-5 Full (KSADS-5) | Single Item |
| ksads_back_grade_repeat | Child Report: Has child repeated a grade? | Diagnostic Interview for DSM-5 Full (KSADS-5) | Single Item |
| ksads_back_sex_orient | Child Report: Child sexual orientation | Diagnostic Interview for DSM-5 Full (KSADS-5) | Single Item |
| ksads_back_sex_orient_probs | Child Report: Does child have problems from being gay? | Diagnostic Interview for DSM-5 Full (KSADS-5) | Single Item |
| ksads_back_trans_id | Child Report: Is child transgender? | Diagnostic Interview for DSM-5 Full (KSADS-5) | Single Item |
| ksads_back_trans_prob | Child Report: Does child have problems from being transgender? | Diagnostic Interview for DSM-5 Full (KSADS-5) | Single Item |
| ksads_bipolar_composite | Child Report: Bipolar Diagnosis | Diagnostic Interview for DSM-5 Full (KSADS-5) | Multi-item scale |
| ksads_cd_composite | Child Report: Conduct Disorder Diagnosis | Diagnostic Interview for DSM-5 Full (KSADS-5) | Multi-item scale |
| ksads_depressive_comp | Child Report: Depressive Disorder Diagnosis | Diagnostic Interview for DSM-5 Full (KSADS-5) | Multi-item scale |
| ksads_eating_disorder_composite | Child Report: Eating Disorder Diagnosis | Diagnostic Interview for DSM-5 Full (KSADS-5) | Multi-item scale |
| ksads_GAD_composite | Child Report: Generalized Anxiety Diagnosis | Diagnostic Interview for DSM-5 Full (KSADS-5) | Multi-item scale |
| ksads_nssi_composite | Child Report: Non-Suicidal Self Injury | Diagnostic Interview for DSM-5 Full (KSADS-5) | Multi-item scale |
| ksads_OCD_composite | Child Report: Obsessive Compulsive Diagnosis | Diagnostic Interview for DSM-5 Full (KSADS-5) | Multi-item scale |
| ksads_psychosis_composite | Child Report: Psychotic Disorder Diagnosis | Diagnostic Interview for DSM-5 Full (KSADS-5) | Multi-item scale |
| ksads_ptsd_composite | Child Report: Post Traumatic Stress Diagnosis | Diagnostic Interview for DSM-5 Full (KSADS-5) | Multi-item scale |
| ksads_SAD_composite | Child Report: Social Anxiety Diagnosis | Diagnostic Interview for DSM-5 Full (KSADS-5) | Multi-item scale |
| ksads_sud_composite | Child Report: Substance Use Disorder Diagnosis | Diagnostic Interview for DSM-5 Full (KSADS-5) | Multi-item scale |
| ksads_suicide_composite | Child Report: Suicide ideation, intent, or attempt | Diagnostic Interview for DSM-5 Full (KSADS-5) | Multi-item scale |
| lmt_scr_avg_rt | Average Response time on Little Man Task | Little Man Task | Score From Task |
| lmt_scr_efficiency | Efficiency on Little Man Task | Little Man Task | Score From Task |
| lmt_scr_perc_correct | Percent Correct on Little Man Task | Little Man Task | Score From Task |
| macvs_ss_fo_p | Mexican American Values: Family Obligation | Mexican American Cultural Values Scale Modified (MACV) | Multi-item scale |
| macvs_ss_fr_p | Mexican American Values: Family as a Referent | Mexican American Cultural Values Scale Modified (MACV) | Multi-item scale |
| macvs_ss_fs_p | Mexican American Values: Family Support | Mexican American Cultural Values Scale Modified (MACV) | Multi-item scale |
| macvs_ss_isr_p | Mexican American Values: Independence & Self-Reliance | Mexican American Cultural Values Scale Modified (MACV) | Multi-item scale |
| macvs_ss_r_p | Mexican American Values: Religion | Mexican American Cultural Values Scale Modified (MACV) | Multi-item scale |
| married.bl | Parent Married | Demogarphic | Single Item |
| medhx_er_composite | Number of emergency room visits for child | Medical History Questionnaire | Multi-item scale |
| meim_ss_total_p | Identification with Ethnicity | Multi-Group Ethnic Identity-Revised Survey (MEIM) | Multi-item scale |
| neighb_phenx_ss_mean_p | Neighborhood Safety | Neighborhood Safety/Crime Survey Modified from PhenX (NSC) | Multi-item scale |
| neurocog_cash_choice_task | Simple Measure of Delay Discounting | Cash Choice Task | Single Item |
| nihtbx_cardsort_uncorrected | Raw Score on NIH Toolbox Card Sort Task | NIH Toolbox | Score From Task |
| nihtbx_flanker_uncorrected | Raw Score on NIH Toolbox Flanker Task | NIH Toolbox | Score From Task |
| nihtbx_list_uncorrected | Raw Score on NIH Toolbox List Sort Task | NIH Toolbox | Score From Task |
| nihtbx_pattern_uncorrected | Raw Score on NIH Toolbox Pattern Recognition Task | NIH Toolbox | Score From Task |
| nihtbx_picture_uncorrected | Raw Score on NIH Toolbox Picture Recognition Task | NIH Toolbox | Score From Task |
| nihtbx_picvocab_uncorrected | Raw Score on NIH Toolbox Picture Vocabulary Task | NIH Toolbox | Score From Task |
| nihtbx_reading_uncorrected | NIH Toolbox Reading Task | NIH Toolbox | Score From Task |
| parental_monitoring_ss_mean | Parental Monitoring of Child | Parental Monitoring Survey | Multi-item scale |
| pea_wiscv_trs | Raw Score on WISC Matrix Reasoning | Weschler Intelligence Scale for Children V | Score From Task |
| physical_activity_1 | Physical Activity | Youth Risk Behavior Survey Exercise Physical Activity (YRB) | Single Item |
| prodrom_psych_ss_severity_score | Prodromal Symptoms of Schizophrenia | Prodromal Psychosis Scale | Multi-item scale |
| prosocial_ss_mean | Prosocial Behavior Child Report | Prosocial Behavior Survey | Multi-item scale |
| prosocial_ss_mean_p | Prosocial Behavior Parent Report | Prosocial Behavior Survey | Multi-item scale |
| prq_q1_p | Parent Rules about drinking | Parental Rules on Substance Use | Single Item |
| puberty | Average of parent & child: Pubertal Development | Demographic | Single Item |
| race.6level | Race | Demographic | Single Item |
| resiliency_num_friends_cat | Number of Friends | Resilience Survey | Single Item |
| resiliency_opposite_sex_friends_cat | Number of opposite sex friends | Resilience Survey | Single Item |
| resiliency_same_sex_friends_cat | Number of same sex friends | Resilience Survey | Single Item |
| school_risk_phenx_ss_dfs | School Disengagement | School Risk and Protective Factors Survey | Multi-item scale |
| school_risk_phenx_ss_iiss | School Involvement | School Risk and Protective Factors Survey | Multi-item scale |
| school_risk_phenx_ss_ses | School Environment Subscale | School Risk and Protective Factors Survey | Multi-item scale |
| screentime_ss_weekday | Weekday Screen Time Child Report | Screen Time Survey (STQ) | Multi-item scale |
| screentime_ss_weekend | Weekend Screen Time Child Report | Screen Time Survey (STQ) | Multi-item scale |
| screentime_week_p | Weekday Screen Time Parent Report | Screen Time Survey (STQ) | Multi-item scale |
| screentime_weekend_p | Weekend Screen Time Parent Report | Screen Time Survey (STQ) | Multi-item scale |
| sex | Biological Sex | Demographic | Single Item |
| sleep_ss_total_p | Sleep Disturbances | Sleep Disturbance Scale for Children (SDS) | Multi-item scale |
| sports_activity_activities_p_hobbies | Participation in Hobbies (e.g., stamp collecting) | Sports and Activities Involvement Questionnaire (SAIQ) | Multi-item scale |
| sports_activity_activities_p_ind_sport | Participation in Individual Sports (e.g., tennis) | Sports and Activities Involvement Questionnaire (SAIQ) | Multi-item scale |
| sports_activity_activities_p_performance | Participation in Performance Sports (e.g., dance) | Sports and Activities Involvement Questionnaire (SAIQ) | Multi-item scale |
| sports_activity_activities_p_team_sport | Participation in Team Sports (e.g., baseball) | Sports and Activities Involvement Questionnaire (SAIQ) | Multi-item scale |
| su_caff_ss_sum_calc | Caffeine consumption | Substance Use Questionnaire | Single Item |
| su_crpf_avail_sum | Substances available to child | Substance Use Questionnaire | Single Item |
| su_tlfb_cal_scr_num_events | Child past substance Use | Substance Use Questionnaire | Single Item |
| upps_ss_lack_of_perseverance | UPPS Lack of perseverance | UPPS Impulsivity Scale | Multi-item scale |
| upps_ss_lack_of_planning | UPPS Lack of Planning/Premeditation | UPPS Impulsivity Scale | Multi-item scale |
| upps_ss_negative_urgency | UPPS Negative Urgency | UPPS Impulsivity Scale | Multi-item scale |
| upps_ss_positive_urgency | UPPS Positive Urgency | UPPS Impulsivity Scale | Multi-item scale |
| upps_ss_sensation_seeking | UPPS Sensation Seeking | UPPS Impulsivity Scale | Multi-item scale |
| via_accult_ss_amer_p | Vancouver Acculturation Index American Culture Scale | Vancouver Index of Acculturation-Short Survey (VIA) | Multi-item scale |
| via_accult_ss_hc_p | Vancouver Acculturation Index Heritage Culture Scale | Vancouver Index of Acculturation-Short Survey (VIA) | Multi-item scale |

**Supplemental Table 3.** Variables used in benchmarks that were not included in descriptive analyses. These variables were excluded from descriptive analyses because of their overlap with other variables in the descriptive analyses (e.g., total IQ not included in descriptive analyses because each of its constituent scales was included).

| ABCD MEASURE ID | DESCRIPTION OF VARIABLE | INSTRUMENT | TYPE |
| --- | --- | --- | --- |
| cbcl_scr_syn_totprob_r | Child Total Psychiatric Problems | Child Behavior Checklist | Multi-item scale |
| asr_scr_totprob_r | Parent Total Psychiatric Problems | Achenbach Self Report, Parent | Multi-item scale |
| nihtbx_totalcomp_uncorrected | Total IQ/Total Cognitive Function | NIH Toolbox | Score From Task |
| nihtbx_fluidcomp_uncorrected | Fluid Intelligence | NIH Toolbox | Score From Task |
| nihtbx_cryst_uncorrected | Crystallized Intelligence | NIH Toolbox | Score From Task |

**Supplemental Table 4.** Detailed procedure on analyses testing relative importance of grouping schemes.

A) step-by-step description of procedure and accompanying R code.

| **Step** | **Procedure** | **R Code** |
| --- | --- | --- |
| 1 | Upload a dataframe of all effect sizes from ABCD dataset (unit Pearson’s correlation) | data = read.csv("/home/max/Documents/  DRD/owens_updated.csv") |
| 2 | Convert Pearson’s correlation to a log transformed z-score | z = (log(FisherZ(r) * (sqrt(n-3))) |
| 3 | Run regression testing within/between instrument as a predictor | lm(z ~ within_instrument) |
| 4 | Run regression testing within/between domain as a predictor | lm(z ~ within_domain) |
| 5 | Run regression testing within/between reporter as a predictor | lm(z ~ within_reporter) |
| 6 | Run regression testing within/between instrument + domain + reporter as predictors | lm(z_log ~ within_domain + within_instrument + within_reporter) |

B) Example of final dataframe used in procedure.

| **Row Index** | **Pearson’s R** | **Z-scored Pearson’s R** | **Within Instrument** | **Within Domain** | **Within Reporter** |
| --- | --- | --- | --- | --- | --- |
| 1 | .03 | Zscore(.03) | 1 | 0 | 1 |
| 2 | .1 | Zscore(.1) | 1 | 1 | 1 |
| 3 | .05 | Zscore(.05) | 0 | 0 | 0 |
| 4 | .00 | Zscore(.00) | 0 | 1 | 0 |
| … | …. | … | … | … | … |
| 12,880 | .04 | Zscore(.04) | 0 | 1 | 1 |

**Supplemental Table 5.** Measures for which it was possible to calculate Crohnbach’s alpha (*n* = 53).

| Variable Names | Cronbach’s Alpha |
| --- | --- |
| asr_scr_aggressive_r | **0.8** |
| asr_scr_anxdep_r | **0.88** |
| asr_scr_attention_r | **0.84** |
| asr_scr_intrusive_r | **0.61** |
| asr_scr_perstr_r | **0.72** |
| asr_scr_rulebreak_r | **0.68** |
| asr_scr_somatic_r | **0.79** |
| asr_scr_thought_r | **0.66** |
| asr_scr_withdrawn_r | **0.75** |
| bisbas_ss_bas_drive | **0.77** |
| bisbas_ss_bas_fs | **0.66** |
| bisbas_ss_bas_rr | **0.73** |
| cbcl_scr_07_ocd_r | **0.69** |
| cbcl_scr_07_sct_r | **0.61** |
| cbcl_scr_07_stress_r | **0.81** |
| cbcl_scr_syn_aggressive_r | **0.89** |
| cbcl_scr_syn_anxdep_r | **0.81** |
| cbcl_scr_syn_attention_r | **0.86** |
| cbcl_scr_syn_rulebreak_r | **0.7** |
| cbcl_scr_syn_social_r | **0.74** |
| cbcl_scr_syn_somatic_r | **0.68** |
| cbcl_scr_syn_thought_r | **0.69** |
| cbcl_scr_syn_withdep_r | **0.73** |
| crpbi_acceptance_ss_studycaregiver | **0.71** |
| ehi_ss_score | **0.86** |
| fes_ss_fc | **0.68** |
| fes_ss_fc_p | **0.66** |
| macvs_ss_fo_p | **0.72** |
| macvs_ss_fr_p | **0.7** |
| macvs_ss_fs_p | **0.8** |
| macvs_ss_isr_p | **0.61** |
| macvs_ss_r_p | **0.97** |
| meim_ss_total_p | **0.89** |
| neighb_phenx_ss_mean_p | **0.88** |
| prodrom_psych_ss_severity_score | **0.86** |
| prosocial_ss_mean | **0.58** |
| prosocial_ss_mean_p | **0.8** |
| school_risk_phenx_ss_dfs | **0.2** |
| school_risk_phenx_ss_iiss | **0.65** |
| school_risk_phenx_ss_ses | **0.61** |
| sleep_dist_da | **0.57** |
| sleep_dist_dims | **0.73** |
| sleep_dist_does | **0.72** |
| sleep_dist_sbd | **0.37** |
| sleep_dist_swtd | **0.59** |
| sleep_ss_total_p | **0.83** |
| upps_ss_lack_of_perseverance | **0.7** |
| upps_ss_lack_of_planning | **0.73** |
| upps_ss_negative_urgency | **0.63** |
| upps_ss_positive_urgency | **0.77** |
| upps_ss_sensation_seeking | **0.49** |
| via_accult_ss_amer_p | **0.9** |
| via_accult_ss_hc_p | **0.91** |

**Supplemental Table 6.** Effect size in Spearman’s rho quantiles for all applicable analytic variations.

| Quantile | .1 | .25 | .5 | .75 | .9 |
| --- | --- | --- | --- | --- | --- |
| Basic | 0.00 | 0.01 | 0.03 | 0.07 | 0.14 |
| Within Instrument | 0.01 | 0.02 | 0.05 | 0.14 | 0.37 |
| Between Instrument | 0.00 | 0.01 | 0.03 | 0.07 | 0.13 |
| Within Reporter | 0.01 | 0.01 | 0.04 | 0.10 | 0.20 |
| Between Reporter | 0.00 | 0.01 | 0.03 | 0.06 | 0.11 |
| Within Domain | 0.01 | 0.02 | 0.06 | 0.15 | 0.29 |
| Between Domain | 0.00 | 0.01 | 0.03 | 0.06 | 0.11 |
| *p* <.05 | 0.02 | 0.03 | 0.06 | 0.10 | 0.17 |
| FDR | 0.03 | 0.04 | 0.06 | 0.10 | 0.17 |
| Bonferroni | 0.05 | 0.06 | 0.09 | 0.14 | 0.22 |
| Partial | 0.01 | 0.01 | 0.04 | 0.10 | 0.22 |

**Supplemental Figure 1.** Distribution of Chronbach’s alphas (*n* = 53).


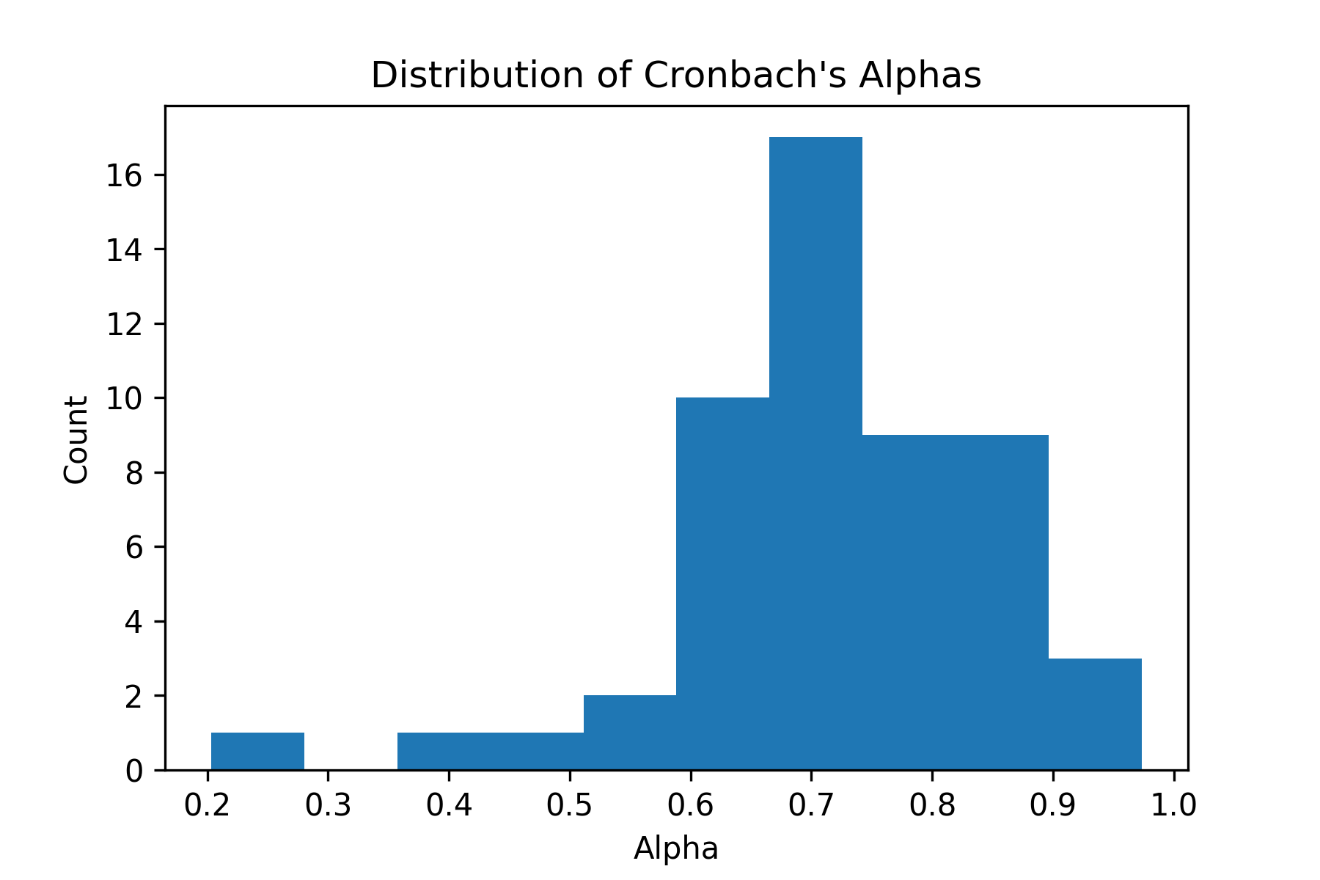

Supplement: S1 File — (DOCX) [file pone.0257535.s001.docx]
